# Hemodynamic changes associated with neuraxial anesthesia in pregnant women with covid 19 disease: a retrospective case-control study

**DOI:** 10.1186/s12871-022-01719-0

**Published:** 2022-06-09

**Authors:** D. Sangroula, B. Maggard, A. Abdelhaleem, S. Furmanek, V. Clemons, B. Marsili, R. Stikes, M. Hill, A. Sigdel, S. P. Clifford, J. Huang, O. Akca, M. C. Logsdon

**Affiliations:** 1 Department of Anesthesiology & Perioperative Medicine, University of Louisville School of Medicine, 530 S. Jackson Street, Room C2A03, Louisville, KY 40202 USA; 2 Center of Excellence for Research in Infectious Diseases, University of Louisville School of Medicine, 501 East Broadway, Suite 100, Louisville, KY 40202 USA; 3 Division of Infectious Diseases, University of Louisville School of Medicine, 501 East Broadway, Suite 100, Louisville, KY 40202 USA; 4 Center for Women, and Infants, University of Louisville Hospital, 530 S. Jackson Street, Louisville, KY 40202 USA; 5 Department of Surgery, University of Louisville School of Medicine, 530 S. Jackson, Louisville, KY 40202 USA; 6 Department of Anesthesiology and Critical Care Medicine, Johns Hopkins University, Baltimore, MD 21218 USA; 7 School of Nursing, University of Louisville, 555 South Floyd Street, Louisville, KY 40292USA USA

**Keywords:** COVID-19, Parturient, Neuraxial Anesthesia, Hemodynamic

## Abstract

**Background:**

Neuraxial blocks is the recommended mode of analgesia and anesthesia in parturients with Coronavirus 19 (COVID-19). There is limited data on the hemodynamic responses to neuraxial blocks in COVID-19 patients. We aim to compare the hemodynamic responses to neuraxial blocks in COVID-19 positive and propensity-matched COVID-19 negative parturients.

**Methods:**

We conducted retrospective, cross-sectional case–control study of hemodynamic changes associated with neuraxial blocks in COVID-19 positive parturients in a Tertiary care academic medical center. Fifty-one COVID-19 positive women confirmed by nasopharyngeal reverse transcription–polymerase chain reaction (RT-PCR), were compared with propensity-matched COVID negative controls (*n* = 51). Hemodynamic changes after neuraxial block were recorded by electronic medical recording system and analyzed using paired and unpaired T- test and Wilcoxon-Mann–Whitney Rank Sum tests. The primary outcome was ≥ 20% change in MAP and HR after neuraxial block placement.

**Results:**

In the epidural group, 7% COVID-19 positive parturients had > 20% decrease in mean arterial pressure (MAP) from baseline compared to 15% COVID-19 negative parturients (*P* = 0.66). In the spinal group, 83% of COVID-19 positive parturients had a decrease in MAP more than 20% from baseline compared to 71% in control (*P* = 0.49). MAP drop of more than 40% occurred in 29% COVID positive parturients in the spinal group versus 17% in COVID-19 negative parturients (*P* = 0.5465). In COVID-19 positive spinal group, 54% required vasopressors whereas 38% in COVID-19 negative spinal group required vasopressors (*P* = 0.387). We found a significant correlation between body mass index (BMI) > 30 and hypotension in COVID ( +) parturient with odds ratio (8.63; 95% CI-1.93 – 37.21) (*P* = 0.007).

**Conclusion:**

Incidence and severity of hypotension after neuraxial blocks were similar between COVID-19 positive and COVID-19 negative parturients. BMI > 30 was a significant risk factor for hypotension as described in preexisting literature, this correlation was seen in COVID-19 positive parturients. The likely reason for parturients with BMI > 30 in COVID negative patients not showing similar correlation, is that the sample size was small.

**Supplementary information:**

The online version contains supplementary material available at 10.1186/s12871-022-01719-0.

## Introduction

According to the Center for Disease Control and Prevention (CDC), pregnant women are at an increased risk for severe illness from Coronavirus Disease 2019 (COVID-19) compared to non-pregnant women and may have an increased risk of adverse pregnancy outcomes [1]. Recommendations for the management of COVID-19 positive parturients by the Society of Obstetric Anesthesia and Perinatology (SOAP) and American College of Obstetricians and Gynecologists (ACOG) include early epidural catheter placement for pain relief in labor to avoid emergency cesarean section (CS) requiring airway instrumentation, which may lead to the spread of the SARS-CoV-2 virus [2–4]. Furthermore, the use of nitrous oxide (N_2_O) for pain relief is avoided in this population due to the concerns of aerosolization of viral particles [2–4]. Remifentanil, a short acting potent narcotic analgesic, is avoided because of potential respiratory depression [2–4]. In addition, general anesthesia (GA) is avoided for concerns of aerosolization of the virus and the potential of positive pressure ventilation worsening lung disease in parturients with COVID-19 disease [2–4].

Even with low dose epidural anesthesia (EA), a sympathetic block may result in vasodilatation in the lower limbs and reduction of venous return. In 10% to 20% of cases, hypotension occurs within one hour following initiation of EA during labor resulting in reduced placental perfusion and oxygen delivery to the fetus [5]. A prolonged drop in placental perfusion pressure may alter maternal–fetal gas exchange, induce fetal heart rate (FHR) abnormalities, and theoretically increase the risk of fetal acidosis [6]. In addition, during the second and third trimesters, the growing uterus actively compresses the inferior vena cava, and results in an overall reduction of effective circulating blood volume [7]. Hemodynamic changes associated with neuraxial blocks can be mitigated by the co-loading of crystalloid and colloid, careful dosing of local anesthetic (LA), use of vasopressors preferably as infusion, uterine displacement, and compression devices [8–10].

In pregnant women hospitalized with pneumonia, maternal fever and hypoxemia may contribute to the development of obstetric complication like preterm labor, preterm premature rupture of the membrane, and abnormal fetal heart rate patterns [11]. Preeclampsia-like symptoms have been described in pregnant women with severe COVID-19 infection that are not preeclamptic [12]. Spinal and epidural anesthesia is associated with less frequent and less severe hypotension in preeclamptic parturients and require smaller doses of vasopressors than normotensive controls with term and preterm pregnancy [13, 14]. Cervical exams are relatively contraindicated in parturients with premature rupture of membranes, which limits the ability to determine onset of labor, regular painful contracton and 2 requests for analgesics have been used as indicator to allow labor epidural [15].

Neuraxial anesthetics may have a significant impact on the hemodynamics of COVID-19 positive parturients. Chen et al. presented a case series of 17 parturients with COVID-19 who had CS, 14 under EA and 3 under GA. These parturients were either asymptomatic or had only mild symptoms without hypoxemia or supplemental oxygen requirement. Under neuraxial block, 12 of the 14 COVID-19 positive parturients experienced severe hypotension, which did not improve with fluid boluses, left uterine tilt, and vasopressors. The authors propose the exaggerated hypotensive response to neuraxial block is related to angiotensin converting enzyme (ACE) II receptor binding of SARS-Cov-2 creating instability in the circulatory system [16]. Although this exaggerated hypotensive response has inspired development of clinical protocols for the anesthetic management of COVID-19 positive parturients, [17] more recent literature has questioned Chen et al.’s conclusions as standard of care hypotension prevention measures for parturients were not practiced as part of the study [18–20].

COVID 19 is related to potential numerous adverse pregnancy related outcomes including significantly higher primary and prelabor caesarean delivery rates. Maternal chronic disease, obstetric complication, severe/critical COVID-19 infection, ICU admission, oxygen support, tocolysis, antenatal corticosteroid prophylaxis, induction failure, NICU admission, postpartum worsening in maternal condition and antibiotic use were higher in the covid positive primary cesarean section groups [21]. There are reports of worse manifestation in pregnant women infected by new SARS-COV2 variant. Increased rate of oxygen support requirement and ICU admission has been reported among cases with pneumonia during Alpha variant period compared to the wild type period [22]. Though there are numerous publications highlighting COVID 19 related adverse outcome in parturients, we observed paucity of literature related to the cardiovascular effects associated with neuraxial block. Therefore, we aim to study hemodynamic responses from spinal and epidural anesthesia in COVID-19 positive pregnant women and compare them to propensity-matched COVID-19 negative parturients.

## Methods

The Human Studies Committee of the University of Louisville approved this retrospective case–control study and waived individual participant informed consent (IRB #20.0673). Institutional safeguards to maintain privacy, data security and risk were well detailed, including de-identification of patient information. All the methods were carried out in accordance with relevant guidelines and regulations. The research involved minimal to no risk to the participants.

### Study population

We conducted a retrospective, cross sectional, case control study of hemodynamic changes associated with neuraxial blocks in COVID-19 positive parturients. COVID-19 status was determined by Reverse transcriptase polymerase chain reaction (RT-PCR) of nasopharyngeal swabs. Patient information regarding demographics and past medical history as well as specifics of the anesthetic procedures were collected from the patient’s medical records. Patients included for study were parturients admitted between 4/1/2020 and 12/30/2020 that tested positive for COVID-19 at the time of delivery and received neuraxial anesthesia for labor pain and/or CS. Parturients requiring replacement of an epidural catheter due to inadequate pain control and those with failed or inadequate spinal blockade (thoracic level, T4-T6) requiring conversion to general anesthesia were excluded. The control group included 51 parturients identified from the University of Louisville Obstetric Database that delivered during the same period (pool of about 1200 parturients). The COVID-19 positive and negative control groups were propensity matched by the following factors: age, gestational age, co-morbidities, mode of delivery, and type of anesthesia.

### Measurements

#### Perioperative/Periprocedural management

All study parturients received anesthetic and obstetric management according to standard practices. For epidural placement, this included a crystalloid bolus of 500 ml through a peripheral IV at the time of procedure. Parturients were monitored from their baseline (before neuraxial block) to the end of the labor. Monitoring included heart rate (HR), electrocardiogram (EKG), respiratory rate (RR), noninvasive systolic and diastolic blood pressure (SBP & DBP), mean blood pressure (MAP), and fingertip pulse oximetry (SpO2). Fetal heart rate (FHR) and uterine contractions were recorded electronically by nursing staff.

The epidural space was identified with loss of resistance technique using saline. A solution containing 3 mL of 1.5% lidocaine with 5 µg/mL of epinephrine was used as a test dose to rule out intravascular and intrathecal injections. A 6–10 ml bolus of premixed epidural solution of 0.1% bupivacaine with 2 µg/mL fentanyl was subsequently injected in incremental doses. The parturient was positioned with uterine displacement. Continuous monitoring of HR, RR, and SpO2 was conducted, whereas BP was recorded every 2 min for the first 20 min and every 15 min for the entire labor period following the first 20 min. After initial dosing of the epidural catheter, a patient controlled epidural anesthesia (PCEA) infusion pump was programmed and initiated with the following parameters: basal rate of 8-10 ml/hr., PCEA bolus of 5 ml with a 15-min lockout interval. Parturients with breakthrough pain were treated with 5 ml of 2% lidocaine bolus, 0.25% bupivacaine bolus, or 5 ml bolus from the epidural pump as per anesthesia provider’s preference. Hemodynamic variables during the first hour following initiation of the epidural anesthesia were used for comparison in this study.

For CS, a 700-1000 ml IV bolus of crystalloid was administered. Spinal anesthesia was subsequently initiated with 1.4–1.6 mL of 0.75% hyperbaric bupivacaine with 10–15 µg of fentanyl and 100-150mcg micrograms of morphine administered in the subarachnoid space. Bupivacaine dosage was determined using patient height. The sensory level of the block was assessed, and the CS was started once a dermatomal level of T4-T6 was achieved. Blood pressure was cycled every 3 min for the entirety of the case unless more frequent monitoring was warranted. Blood loss and Apgar scores were recorded. Hemodynamic changes after placement of the neuraxial block were identified from the anesthesia intraoperative record and nursing record. An electronic medical recording system was used to capture intraoperative vitals.

#### Definition of Hypotension & Bradycardia

Maternal hypotension was defined as any recorded MAP less than baseline and/or any requirement for vasopressor therapy. Hypotension was categorized into the following groups based on the decrease in MAP from baseline: less than 20%, 20–30%, 30–40%, and greater than 40% from baseline. Maternal bradycardia was defined as a heart rate less than 60 beats/min or a greater than 20% decrease from baseline heart rate. In addition, FHR abnormalities, mode of delivery, and 1- and 5-min Apgar scores were recorded. Total fluid administered as well as vasopressor and uterotonic usage were also recorded for patients that delivered by CS. Any major adverse event during or after the procedure was identified.

#### Sample size

A sample-size estimate was determined based upon the primary outcome. Specifically for the spinal anesthesia group, the intention was to detect a 20% difference in MAP (MAP 85 vs. 68 mmHg; with a standard deviation (SD) of 20 mmHg), *p*-value (alpha) < 0.05, and 80% power. We calculated a minimum of 44 parturients (*n* = 22 per each group) to complete the study.

#### Data Collection, Propensity Matching & Data Analysis

We identified 51 parturients that tested positive for COVID-19 at an academic health sciences center between 4/1/2020 and 12/30/2020. These COVID -19 positive parturients served as the “study group”. We accessed the database of ~ 1200 parturients from the same period that tested negative for COVID-19 in order to identify a propensity matched “control group”. The control group was selected after propensity matching for the following variables: age, hypertension, gestational diabetes, gestational age, anemia, maternal age, type of neuraxial block, and mode of delivery. A total of 51 COVID-19 positive cases and 51 propensity-matched negative controls were included in the final analysis. Of the 51 COVID-19 positive parturients, 27 received epidural analgesia and 24 received spinal anesthesia. Hemodynamic data of parturients who received spinal and epidural anesthetics were analyzed separately.

Outcomes were compared between the groups with unpaired, two-tailed t-tests. Non-normally distributed results were analyzed by Wilcoxon-Mann–Whitney Rank Sum tests. Results were presented as means ± standard deviations, medians (95% confidence intervals), actual values, or percentages. A *p*-value < 0.05 was considered statistically significant. The primary outcome was ≥ 20% change in MAP and HR after neuraxial block placement. Secondary outcomes were as follows: new FHR change after epidural placement, CS requirement, or instrumental delivery secondary to FHR changes, uterotonic requirement, incidence of side effects from epidural and drug delivery, blood loss, duration of hospital stay, and the incidence of Apgar score < 7 at 5 min.

## Results

Demographics of COVID-19 positive and negative parturients were similar in terms of age, BMI, weight, gestational age, and co-morbidities (i.e., propensity matched samples). Statistically significant differences were seen between the heights of COVID positive and COVID negative parturients (*p* = 0.048) (See Table 1). Of the 51 COVID-19 positive parturients, 54% (27/51) had hypertension. Eleven (22%) of the COVID-19 positive parturients presented with gestational hypertension, 9 (18%) had pre-eclampsia without severe features, and 7 (14%) had pre-eclampsia with severe features. This was similar to hypertension disorders in the control group 31/51(60%).

**Table 1:**
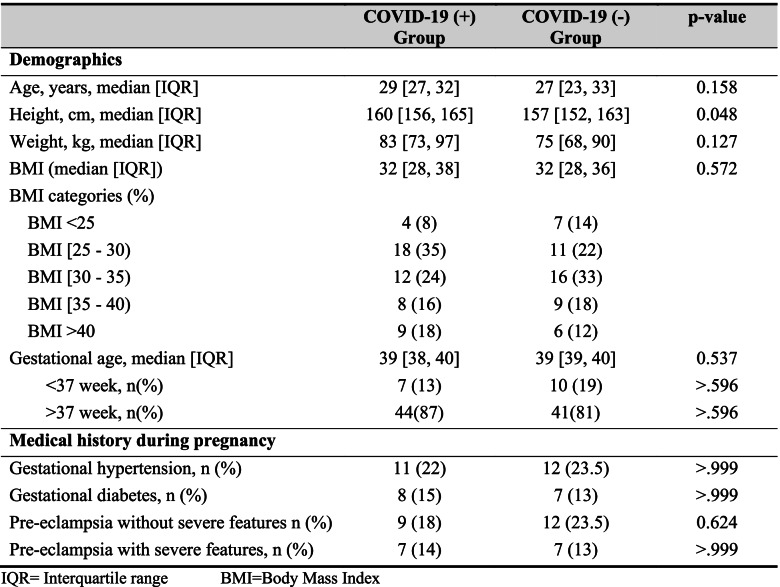
Demographic & morphometric characteristics and baseline comorbidities

Out of 51 COVID-19 positive parturients, 18 (35%) were symptomatic. One woman was symptomatic and required high flow oxygen and ICU (intensive care unit) admission, but the rest of the parturients had mild symptoms. Fever, cough, and fatigue were the most reported symptoms.

The most common indication for CS was history of previous CS. Indications for urgent CS were maternal and fetal indications, which were not directly related to increased oxygen requirements or disease severity of COVID-19. Pre-eclampsia with severe features was the most common indication for urgent CS. Out of 27 parturients who in the COVID positive, epidural group, 3 delivered via CS and 24 had vaginal delivery.

Average blood loss associated with CS was 800 ml in the COVID-19 positive parturients and 600 ml in COVID-19 negative parturients (*P* = 0.11). APGAR scores for each group were also similar at both the 1 min and 5 min assessments. (Table 2). Blood loss in vaginal delivery was similar in both groups. (Table 2).

**Table 2:** Blood loss, APGAR score, intraoperative fluid and uterotonic use during Cesarean section

	COVID 19 ( +) (*n* = 24)	Controls 19 (-) (*n* = 24)	*p*-value
**Blood loss, (ml)** median [IQR]	800.0 [250.0, 400.0]	600.0 [250.0, 400.0]	0.11
** APGAR 5 MIN,** median [IQR]	9.0 [9.0, 9.0]	9.0 [9.0, 9.0]	0.699
** APGAR < 7** (5 min), n (%)	0 (0)	0 (0)	
**Intraoperative fluid,** median [IQR]	1800.0 [1500.0, 2000.0]	1900.0 [1500.0, 2000.0]	0.841
**Uterotonics**			
**Oxytocin,** n (%)	24 (100)	24 (100)	NA
Dose (U), median [IQR]	23.0 [20.0, 40.0]	23.0 [6.0, 27.0]	
**Misoprostol,** n (%)	3 (12.5)	2 (8)	> .999
**Methergine,** n (%)	0 (0)	1 (4)	0.954

*Abbreviations*: *U* Units, *IQR* Interquartile range, *APGAR* Appearance, pulse, grimace, activity, respiration

Usage of uterotonic agents was similar between the two groupings. Oxytocin was used in 100% of parturients in both groups, and second line uterotonics were necessary in 3/24 (12%) parturients from each group. In the COVID-19 positive cohort, 3 parturients were given misoprostol whereas in the control group, 2 parturients received misoprostol and 1 required methergine. (Table 2).

There were not statistically significant hemodynamic differences in COVID-19 positive and negative parturients who received epidural blocks. (Table 3) Baseline MAP was similar in each group (*p* =  > 0.799). Only 2/27 (7%) of the parturients in the COVID-19 positive group had blood pressure drops of > 20% from baseline compared to 4/27 (14%) within the control group (*p* =  > 0.66). There were no new FHR changes associated with the drop in blood pressure and vasopressor intervention was not required.

**Table 3:** Blood pressure and heart rate changes in patients receiving epidural anesthesia

Epidural block (*n* = 54)			
	**COVID-19 ( +) (27)**	**COVID-19(-) (27)**	***p*-value**
**Baseline MAP,** median [IQR]	90.0 [80.0, 99.5]	89.0 [86.5, 96.0]	0.799
**No change in MAP,** n (%)	20 (74)	20 (74)	> .999
**Change in MAP,** n (%)	7 (26)	7 (26)	> .999
** > 20%**	2(7)	4(15)	0.66
20–30%	0 (0)	2 (7)	
30–40%	2 (7)	2 (7)	
> 40%	0 (0)	0 (0)	
**Baseline heart rate,** median [IQR]	88.0 [80.5, 95.5]	90.0 [82.5, 99.5]	0.124
**Decrease in heart rate,** n (%)	7 (26)	5 (19)	0.131
**Percent change in heart rate**			
Less than 20%	5 (19)	1 (4)	
20–30%	2 (7)	4 (15)	
30–40%	0 (0)	0 (0)	
> 40%	0 (0)	0 (0)	
**Increase in heart rate,** n (%)	7 (39)	5 (19)	0.371
**Percent change in heart rate**			
Less than 20%	3 (11)	1 (4)	
20–30%	2 (7)	3 (11)	
30–40%	0 (0)	1 (4)	
**Blood loss, ml** Vaginal **delivery** median [IQR]	300	300	0.99

*Abbreviations*: *MAP* Mean arterial pressure, *ARV* of *MAP* Variability of mean arterial pressure

Regarding parturients who received spinal blocks for CS, baseline blood pressure and heart rates were similar between the COVID positive and negative parturients. For parturients receiving spinal anesthesia, the incidence of MAP decreases > 20% in the COVID-19 positive group was 20/24 (83%), which was similar to the COVID-19 negative group 17/24 (71%, *P* = 0.49). Of note, each group had 5/24 (21%) patients with drop in MAP of 20–30% (*P* =  > 0.99), and 8/24 (33%) with a 30–40% drop in MAP (*p* =  > 0.99). A more profound MAP drop of > 40% was observed in 7/24 (29%) of the COVID-19 positive group compared to 4/24 (17%) in negative controls, although this observation was not statistically significant (*P* = 0.546). (See Table 4.)

**Table 4:** Blood pressure and heart rate changes and vasopressor requirement in patients receiving spinal anesthesia

	**COVID-19 ( +)**	**COVID-19 (-)**	***p*-value**
	**24**	**24**	
**Baseline MAP,** median [IQR]	89.5 [82.0, 104.2]	91.0 [84.0, 101.0]	0.721
**Drop in MAP,** n (%)	23 (96)	23 (96)	> .999
Less than 20%	3 (13)	6 (25)	0.599
More than 20%	20(83)	17(71)	0.49
20–30%	5(21)	5(21)	> 0.99
30–40%	8 (33)	8(33)	> 0.99
> 40%	7(29)	4 (17)	0.546
**ARV**^**2**^** of MAP (variability of MAP),** median [IQR]	163.64 [76.79, 266.66]	124.75 [63.4, 182.62]	0.344
**Baseline heart rate,** median [IQR]	92.5 [81.0, 100.0]	91.0 [80.8, 100.8]	0.974
**Decrease in heart rate,** n (%)	15 (62)	16 (67)	> .999
**Percent decrease in heart rate**			
Less than 20%	4 (17)	2 (8)	
20–30%	6 (25)	7 (29)	0.99
30–40%	3 (12)	6 (25)	
> 40%	2 (8)	1 (4)	
**Increase in heart rate,** n (%)	13 (54)	13 (54)	0.288
**Percent increase in heart rate**			
Less than 20%	4 (17)	4 (17)	
20–30%	5 (21)	5 (21)	
30–40%	1 (4)	3 (12)	
> 40%	3 (12)	1 (4)	
**Vasopressors required,** n (%)	13 (54)	9 (38)	0.387
**Phenylephrine,** *n* (%)	11 (45.8)	10 (42)	
Total dose, median [IQR]	300 [225, 450]	350[225, 550]	
**Ephedrine,** n (%)	7 (29.1)	0 (0)	
Total dose, median [IQR]	10 [10, 15]	0 [0,0]	

*Abbreviations*: *MAP* Mean arterial pressure, *ARV* of *MAP* Variability of mean arterial pressure

**Fig. 1:**
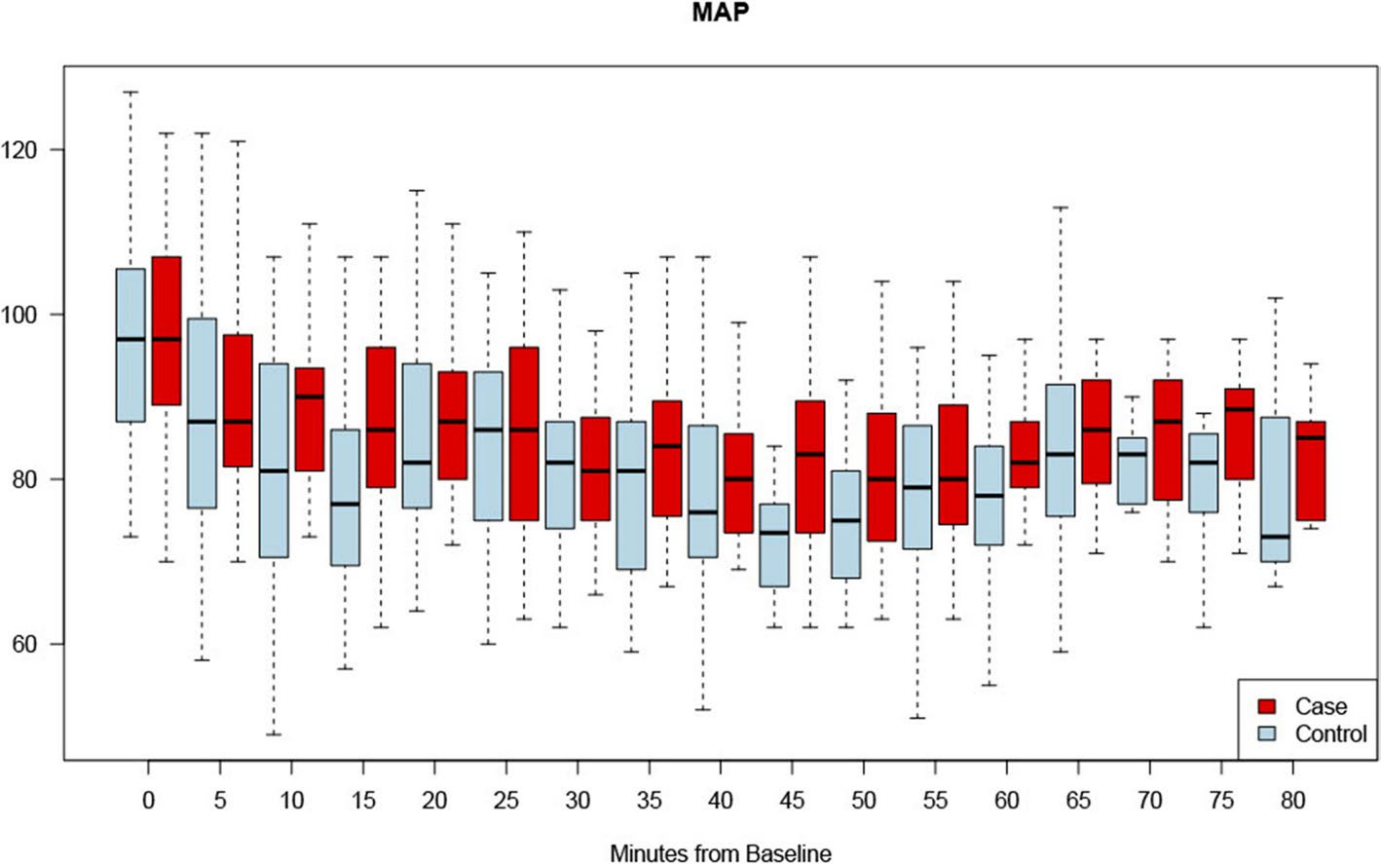
Changes in MAP under spinal anesthesia – blood pressure variability

**Fig. 2:**
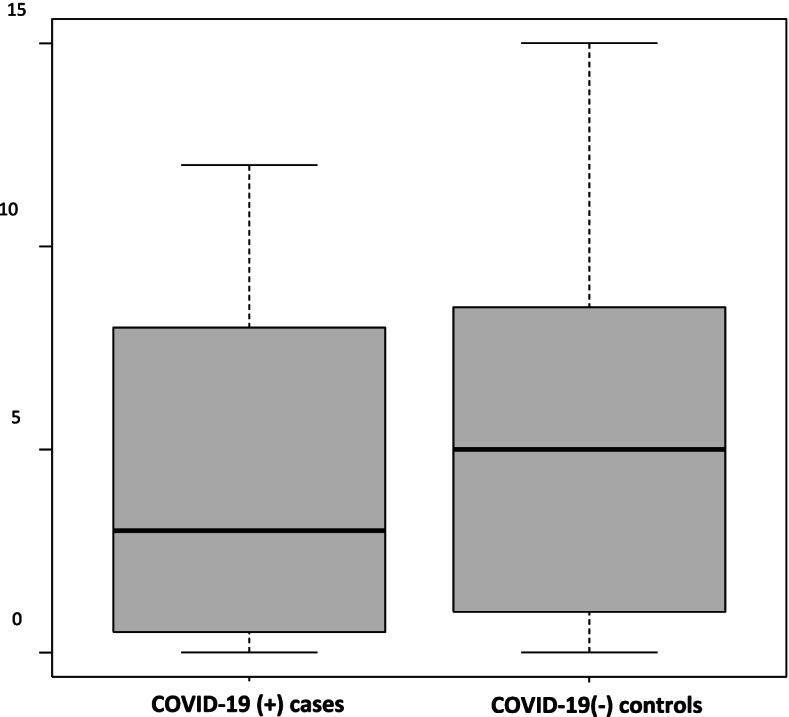
COVID-19 ( +) vs COVID-19 (-) cesarean section: duration of MAP drop > 20% in minutes

**Fig. 3:**
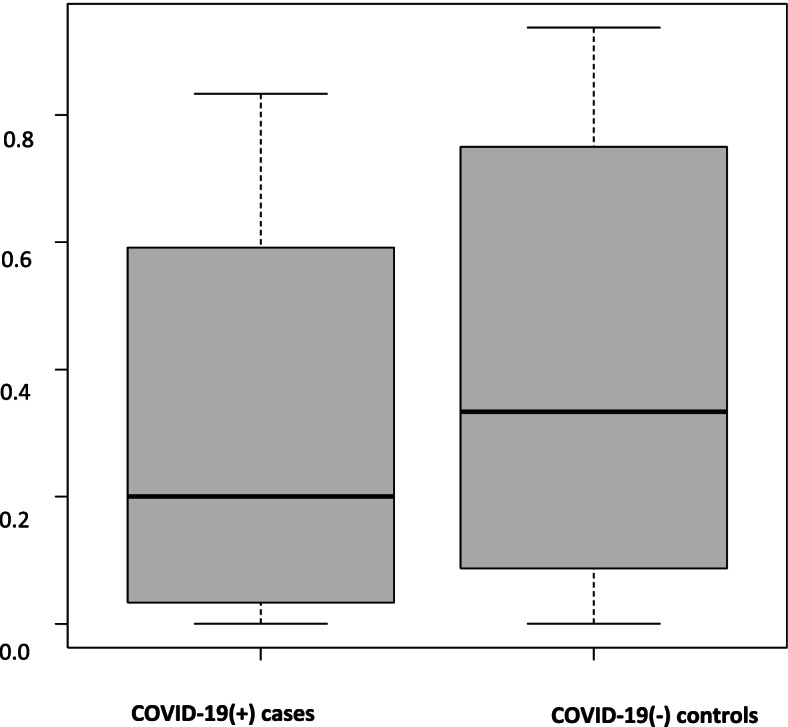
COVID-19 ( +) vs COVID-19 (-) spinal anesthesia comparison: proportion of MAP drop > 20%

There was similar MAP variability in the COVID positive spinal anesthetic group 163.6 (76.7, 266.6) as compared to the negative control group 124.7 (63.4, 182.6) hg(*P* = 0.344) (Table 4 and Fig. 1). The duration of MAP decreases > 20% from baseline was similar in both groups, with a median time of 25 min [inter quartile range (IQR5-42.5 min)] in COVID-19 positive parturients and 25 min (IQR 2.5–40 min) in the control group. (*P* = 0.562) (Table 4, Figs. 2 and 3).

Among COVID-19 positive parturients who underwent CS under spinal anesthesia, 54.1% required vasopressors; 45.8% required phenylephrine and 29% required ephedrine. Only 37.5% required vasopressors in the control group; however, no statistical difference was detected (*P* = 0.387). Intraoperative fluid use was similar in both groups, with the COVID-19 positive group receiving 1800 mL in comparison to 1900 mL in the control group (*p* = 0.841).

Analysis of risk factors for drop in MAP > 20% demonstrated that height, dose, and dermatomal anesthetic levels did not associate with significant decreases in MAP. All parturients in the spinal block group achieved dermatomal level of T4-6. Parturients with higher BMI experienced larger declines in MAP regardless of their COVID-19 status. Patients with BMI > 30 in the COVID-19 positive cohort were more likely to experience a significant drop in MAP with odds ratio (OR) of 8.63 (95% CI-1.93 – 37.21) (*P* = 0.007). However, the propensity score matched COVID-19 negative parturients did not demonstrate this correlation (*P* = 0.106). This association could not be explored in the epidural block groups because only a small percentage of parturients declined > 20% from MAP baselines.

Regarding maternal heart rate, 11/24 (45%) COVID-19 positive parturients receiving spinal anesthesia experienced a decrease > 20% from baseline. This value was similar in the control group 14/24 (58%). Tachycardia was observed equally amongst both groups 13/24 (54%) (Table 4).

## Discussion

In this retrospective, propensity-matched, case-controlled trial assessing COVID-19 positive vs COVID-19 negative parturients receiving epidural or spinal anesthesia, no significant differences were detected in hypotensive events, MAP variability, and HR responses. Obstetric and neonatal outcomes were also similar in both groups. Increased BMI was associated with MAP declines of > 20% from baseline in COVID-19 positive parturients but not in the COVID-19 negative group.

Symptomatic cases in our study presented with fever, dyspnea, fatigue, and cough, which was similar to the presenting signs and symptoms of seven hundred cases hospitalized in seven city hospitals in Louisville, Kentucky [23]. The most common cause of preterm delivery in our COVID-19 positive cohort was preeclampsia with severe features. However, it is noteworthy that preeclampsia-like symptoms have been described in pregnant women with severe COVID-19 infection that are not preeclamptic. This is distinguishable by LDH, uterine artery pulsatility index, and angiogenic factors such as tyrosine kinase-1 and placental growth factor [12]. These tests were not performed in parturients included in our study.

Chen et al. described excessive hypotension in 12/14 parturients who received EA with ropivacaine compared to 3 parturients who received general anesthesia for CS. However, information provided by the authors on the blood pressure trends and vasopressors requirements were limited. In contrast, Qi Zhong et al. reported on 49 COVID-19 positive parturients that had spinal anesthesia with ropivacaine 0.75% for CS and orthopedic surgeries and did not find intraoperative cardiorespiratory compromise or instability in hemodynamics [24].

High incidences of hypertension, hypotension, and tachycardia have been described in parturients with severe COVID-19 infection. ACE2 receptor mediated RAAS dysfunction is believed to be the cause of these cardiovascular manifestations [25]. Atrioventricular block, elevated troponin levels, and pericardial effusion have also been described in severe disease. [26] Breslin et al. reported upon 18 COVID-19 positive obstetric parturients with intrapartum EA, spinal, or combined spinal-epidural anesthesia, and no hemodynamic instability was noted in any of the parturients [27]. In our study, hemodynamic responses were similar between 27 COVID-19 positive parturients and 27 controls utilizing epidural anesthesia for labor. Larger MAP declines were observed in the spinal anesthetic recipients due to the more profound sympathectomy associated with spinal blocks; however, these episodes of hypotension were similar in both COVID-19 positive and negative cohorts.

Of note, COVID-19 positive parturients with BMI > 30 were observed to have higher rates of MAP drops > 20% when compared to COVID-19 positive parturients with BMI < 30. However, COVID-19 negative groups did not demonstrate significant correlation with BMI. Obesity is associated with increased risk of type 2 diabetes, hypertensive disorders of pregnancy, coronary artery disease, congestive heart failure, operative vaginal delivery, post-partum hemorrhage, and fetal macrosomia. Each of these co-morbidities may affect the hemodynamic responses in pregnancy. The potential for higher levels of cephalad sensory blockade in obese patients has been a topic of discussion along with the potential need for dose adjustments in the obese parturient. Studies have reported greater incidences of hypotension in parturients with an elevated BMI, [28, 29] and have recommended decreases in local anesthesia dosing [30]. Additional studies have suggested that obesity did not affect sensory blockade when a smaller volume with dilute LA solution was used [31, 32]. Exaggerated cephalad spread can be explained by a lower volume of cerebrospinal fluid (CSF), which is inversely proportional to increases in BMI, greater compression of the epidural space by engorged epidural veins, and deposition of adipose tissue in the epidural space. In addition to the potential for greater cephalic LA spread, the cardiovascular and hormonal changes seen in all parturients are amplified in the obese parturient population and can compound the possibility of hypotension. The literature remains conflicted. Lee et al., reported ED95 (effective dose in 95% patients) for hyperbaric bupivacaine similar in obese and non-obese parturients with no significant increased sensory block up to a 12 mg dosage [33]. In our study, parturients received up to, but not exceeding 12 mg of bupivacaine in the spinal anesthetic group. Vasculopathy associated with COVID-19 infection could have contributed to increased hypotension in COVID-19 parturients with obesity.

The duration of drop in MAP > 20% from baseline was similar in both groups (25 min), signifying responsiveness to phenylephrine and ephedrine in parturients regardless of their BMI and COVID-19 status. Hypotension was successfully treated with standard doses of phenylephrine and ephedrine unlike the severe hypotension from the previously reported study.^11^ Epidural and spinal blocks were conducted in similar fashions for both groups. For the spinal anesthetics, a sensory dermatomal level of T4-T6 was achieved in both cohorts, indicating comparable levels of sympathectomy. Fluid co-loading and the total volume of fluids administered were also similar. This observation is important as it supports the SOAP and ASA guidelines and further emphasizes the safety of neuraxial anesthesia in the management of COVID-19 positive parturients. To the best of our knowledge, this is the first retrospective case control study reporting on the hemodynamic changes associated with neuraxial anesthesia in COVID-19 positive parturients. Although BMI > 30 was significant to degree of hypotension within the COVID-19 positive parturient group, no observable hemodynamic variances occurred between positive and non-positive parturients receiving neuraxial analgesia, and the obstetric and neonatal outcomes were similar. These finding further support the safety of neuraxial techniques in COVID-19 positive parturients.

Our study has several limitations. Retrospective design without blinding and our relatively small sample size are major limitations. However, propensity score matching provided a more vigorous comparison between COVID-19 positive and negative groups.

In conclusion, incidence, and severity of hypotension after neuraxial blocks were similar between COVID-19 positive and propensity score matched COVID-19 negative parturients. BMI > 30 was a significant risk factor for hypotension in COVID-19 positive parturients, therefore, vigilant monitoring of hemodynamics in COVID-19 positive parturients with BMI > 30 is prudent. Our study demonstrates that neuraxial anesthesia with standard dose of local anesthetic agent, standard practice of monitoring, and vasopressor, is safe and effective anesthetic in COVID-19 positive asymptomatic and mildly symptomatic parturients.

## Supplementary information

**Additional file 1.**

## Data Availability

The datasets used and/or analyzed during the current study are available from the corresponding author on reasonable request. Restrictions apply to the availability of these data.

## Abbreviations

CDC: Center for Disease Control and Prevention
COVID-19: Coronavirus Disease 2019
SOAP: Society of Obstetric Anesthesia and Perinatology
ACOG: American College of Obstetricians and Gynecologists
CS: Cesarean section
N_2_O: Nitrous oxide
GA: General anesthesia
EA: Epidural anesthesia
FHR: Fetal heart rate
LA: Local anesthesia
ACE II: Angiotensin converting enzyme
IRB: Institutional review board
RT-PCR: Reverse transcriptase polymerase chain reaction
HR: Heart rate
EKG: Electrocardiogram
RR: Respiratory rate
SBP: Systolic blood pressure
DBP: Diastolic blood pressure
MAP: Mean blood pressure
SpO2: Pulse oximetry
PCEA: Patient controlled epidural anesthesia
ICU: Intensive care unit
SD: Standard deviation
IQR: Inter quartile range
