# Supplementary material for: Hemodynamic changes associated with neuraxial anesthesia in pregnant women with covid 19 disease: a retrospective case-control study
Source: BMC Anesthesiol. 2022 Jun 9;22:179. doi: 10.1186/s12871-022-01719-0 (PMC9178224; doi:10.1186/s12871-022-01719-0)
Supplement: Supplementary file 1 — Additional file 1. [file 12871_2022_1719_MOESM1_ESM.docx]

**Supplementary Tables**

**Table 6. Risk factors for drops in MAP of 20% or more**

|  | **Odds Ratio** | **95% Confidence**  **Interval** | **p-value** |
| --- | --- | --- | --- |
| **Study group** |  |  |  |
| COVID-19 (-) (baseline) | - | - | - |
| COVID-19 (+) | 1.08 | 0.49 – 2.39 | 0.841 |
|  |  |  |  |
| **Body mass index** |  |  |  |
| < 25 | 4.79 | 0.99 – 25.46 | 0.054 |
| (25 – 30) (baseline) | - | - | - |
| (30 – 35) | 5.75 | 1.68 – 23.59 | 0.008 |
| (35 – 40) | 10.54 | 2.65 – 50.71 | 0.002 |
| ≥ 40 | 8.63 | 2.09 – 42.32 | 0.004 |
|  |  |  |  |
| **Height (10 cm increase)** | 0.79 | 0.45 – 1.38 | 0.415 |
|  |  |  |  |
| **Dermatomal block level** |  |  |  |
| T4 (baseline) | - | - | - |
| T5 or T6 | 1.26 | 0.15 – 27.28 | 0.847 |
|  |  |  |  |
| **Bupivacaine dose (1 mg increase)** | 1.42 | 0.54 – 4.42 | 0.506 |

**Table 7.**

**Correlation between BMI, intrathecal bupivacaine dose and MAP decrease >20%**

**COVID-19 (+) cases, spinal subgroup analysis**

|  | **Odds Ratio** | **95% Confidence**  **Interval** | **p-value** |
| --- | --- | --- | --- |
| **Body mass index** |  |  |  |
| < 30 (baseline) | - | - | - |
| ≥ 30 | 8.63 | 1.93 – 37.21 | 0.007 |
|  |  |  |  |
| **Bupivacaine dose (1 mg increase)** | 1.88 | 0.44 – 12.67 | 0.446 |

**COVID-19 (-) (controls) spinal subgroup analysis**

|  | **Odds Ratio** | **95% Confidence**  **Interval** | **p-value** |
| --- | --- | --- | --- |
| **Body mass index** |  |  |  |
| < 30 (baseline) | - | - | - |
| ≥ 30 | 2.68 | 0.84 – 9.35 | 0.106 |
|  |  |  |  |
| **Bupivacaine dose (1 mg increase)** | 0.95 | 0.23 – 4.51 | 0.994 |
